# When Reduced Myocardial Strain Does Not Mean Myocardial Disease: The Emerging Role of Extrinsic Mechanical Determinants

**DOI:** 10.3390/jcdd13100511

**Published:** 2026-10-10

**Authors:** Andrea Sonaglioni, Gian Luigi Nicolosi

**Affiliations:** 1 Division of Cardiology, IRCCS MultiMedica, 20123 Milan, Italy; 2 Division of Cardiology, Policlinico San Giorgio, 33170 Pordenone, Italy; gianluigi.nicolosi@gmail.com

**Keywords:** myocardial strain, global longitudinal strain, thoracic geometry, antero-posterior thoracic diameter, modified Haller index, pectus excavatum, relative apical sparing

## Abstract

Myocardial strain imaging has become an established tool for detecting subtle abnormalities in cardiac mechanics, and reduced strain is frequently interpreted as evidence of subclinical myocardial dysfunction. However, myocardial deformation reflects not only intrinsic myocardial properties but also the mechanical environment in which the heart operates. Emerging evidence suggests that extracardiac factors, particularly thoracic geometry and reduced antero-posterior (A–P) thoracic dimensions, may be associated with variations in global and regional strain even in individuals without overt structural heart disease. This narrative review examines the evidence supporting an extrinsically mediated myocardial deformation phenotype across different anatomical and physiological models, including pectus excavatum, mitral valve prolapse, healthy pregnancy, and obesity. A recurring feature is the preferential impairment of basal and, to a lesser extent, mid-ventricular deformation with relative preservation of apical mechanics, potentially generating a mechanical phenocopy of relative apical sparing. These abnormalities may reflect true mechanical constraint, measurement-related effects, or their combination, while intrinsic and extrinsic mechanisms may coexist. Thoracic morphometry may therefore provide valuable context when unexpectedly reduced strain is encountered despite preserved conventional cardiac structure and function. The A–P thoracic diameter can be readily measured from the parasternal long-axis view, while the modified Haller index provides a more comprehensive, radiation-free characterization of thoracic conformation. However, the available evidence is predominantly observational, derives in part from selected single-center cohorts, and does not establish a causal relationship between thoracic geometry and myocardial strain abnormalities; accordingly, these morphometric parameters should currently be considered exploratory and not yet validated as diagnostic markers. Rather than equating abnormal strain with abnormal myocardium, myocardial deformation should be interpreted within a multiparametric and mechanistic framework integrating regional strain distribution, conventional cardiac findings, clinical probability, and the mechanical environment surrounding the heart. Accounting for these factors may improve diagnostic specificity and reduce the misclassification of extrinsically mediated strain abnormalities as subclinical myocardial disease, although independent multicenter and multi-vendor validation is required before this hypothesis-generating framework can be incorporated into routine clinical decision-making.

## 1. Introduction

Myocardial strain imaging has become an integral component of contemporary echocardiography, providing a quantitative assessment of myocardial deformation that is more sensitive than conventional measures of ventricular systolic function [1,2,3]. In particular, left ventricular global longitudinal strain (LV-GLS) can detect subtle abnormalities in myocardial mechanics even when left ventricular ejection fraction (LVEF) remains preserved [4,5,6,7,8,9,10]. Consequently, reduced strain magnitude is frequently interpreted as evidence of early or “subclinical” myocardial dysfunction. However, myocardial strain is not a direct measure of intrinsic myocardial contractility, as its assessment may be influenced by multiple physiological, hemodynamic, anatomical, and technical determinants, including loading conditions [11,12], image quality [13], frame-rate settings [14], operator experience [15], and inter-vendor differences in acquisition and post-processing algorithms [16,17]. Accordingly, abnormal myocardial deformation should not invariably be considered synonymous with underlying myocardial disease.

Among the determinants of strain measurements, the role of extracardiac mechanical constraints remains comparatively underrecognized. Thoracic morphology may modify the spatial relationship between the heart, sternum, vertebral column, diaphragm, and surrounding intrathoracic structures, thereby influencing cardiac chamber geometry, myocardial excursion, and regional deformation. In particular, a reduced antero-posterior (A–P) thoracic diameter may limit the space available for normal cardiac motion and preferentially constrain basal myocardial segments [18,19,20,21,22]. This mechanical constraint may promote intraventricular dyssynchrony, contributing not only to reduced global strain values but also to characteristic regional deformation patterns with relative preservation of apical mechanics. Previous studies have suggested that thoracic conformation, as assessed by the modified Haller index (MHI) [23] or by direct measurement of the A–P thoracic diameter, may significantly influence myocardial strain even in individuals without overt structural heart disease.

These observations challenge the conventional assumption that reduced myocardial strain necessarily reflects intrinsic myocardial dysfunction and support the emerging concept of an extrinsically mediated myocardial deformation phenotype. Recognition of this phenotype may be clinically relevant when abnormal global or regional strain is identified in otherwise healthy individuals or in subjects with preserved conventional cardiac structure and function. This narrative review aims to examine the available evidence on the extracardiac and mechanical determinants of myocardial strain impairment, with particular emphasis on thoracic geometry, preferential basal myocardial involvement, relative apical sparing, and the potential role of simple thoracic morphometric assessment in improving the clinical interpretation of strain imaging.

## 2. Myocardial Strain Beyond Intrinsic Contractility: The Role of Extrinsic Mechanical Determinants

Myocardial strain reflects the relative change in myocardial length during the cardiac cycle and is therefore determined not only by intrinsic myocardial contractility but also by the mechanical environment in which the ventricle operates [24,25]. Preload and afterload are well-recognized modulators of myocardial deformation, and changes in ventricular geometry, heart rate, image quality, tracking algorithms, and vendor-specific post-processing may further influence strain measurements. Less attention, however, has been paid to the potential contribution of extracardiac mechanical forces, which may alter myocardial deformation without necessarily implying primary myocardial damage.

The heart is not mechanically isolated within the thoracic cavity. Its motion and deformation occur within a confined anatomical space bounded anteriorly by the sternum and chest wall, posteriorly by the vertebral column, inferiorly by the diaphragm, and laterally by the lungs and mediastinal structures [26]. Consequently, variations in thoracic geometry may modify the space available for cardiac filling, displacement, tilting, rotation, and systolic excursion [27]. A reduced A–P thoracic diameter is particularly relevant because it decreases the sterno-vertebral distance and may increase external constraint on the cardiac chambers [28,29,30].

Thoracic geometry can be quantified non-invasively using the modified Haller index (MHI), calculated as the ratio between the external transverse (L–L) thoracic diameter and the A–P diameter measured from a conventional parasternal long-axis echocardiographic view (MHI = L–L/A–P). The clinical relevance of this anatomical relationship is supported by evidence that an increased MHI, primarily reflecting a reduced A–P chest dimension, is associated with external cardiac compression, smaller chamber dimensions, and altered myocardial mechanics despite the absence of intrinsic myocardial disease [31]. An MHI > 2.5 has been used in previous studies as an exploratory marker of restrictive thoracic conformation; however, this value should not be interpreted as an established diagnostic cut-off, as externally validated reference ranges accounting for age, sex, and body size are currently lacking. Standardization of acquisition remains important. In the originally validated method, the A–P diameter was obtained with the patient in the left lateral decubitus position, with the transducer positioned close to the sternum in the left third or fourth intercostal space to obtain a parasternal long-axis view; the distance was measured from the ultrasound entry point to the anterior surface of the vertebral body, identified using the posterior wall of the descending thoracic aorta as an anatomical landmark [23]. Although a specific relationship between respiratory phase and A–P diameter has not been established, consistent acquisition conditions, including a standardized respiratory phase and avoidance of excessive transducer pressure, appear advisable to improve measurement standardization. Potential influences of body size and sex should also be considered, and dedicated intra- and interobserver reproducibility studies are warranted. The standardized approach to thoracic morphometric assessment is illustrated in Figure 1.

Importantly, the mechanical consequences of thoracic constraint may not be uniformly distributed throughout the myocardium. Available observations suggest that externally mediated deformation abnormalities tend to involve the basal and mid-ventricular segments more prominently than the apex. In infants with pectus excavatum (PE), for example, myocardial deformation impairment was particularly evident at the mid and basal levels, while increased apical rotation was interpreted as a possible compensatory response to basal mechanical compression [32,33]. From a mechanical perspective, this heterogeneous distribution is plausible because different ventricular regions have different degrees of freedom within the thoracic cavity. Basal myocardial segments are anatomically closer to relatively fixed structures and may therefore be more susceptible to external constraint, whereas the apex may retain comparatively greater freedom of motion [34]. Such regional heterogeneity may ultimately generate an apparent base-to-apex deformation gradient, with reduced basal strain and relative preservation of apical deformation [31,32]. The proposed interplay between conventional determinants of myocardial strain, thoracic geometry, extrinsic cardiac constraint, and the resulting regional deformation pattern is schematically summarized in Figure 2.

Several mechanisms may contribute, either individually or synergistically, to this phenomenon. Direct external compression may restrict myocardial excursion; altered cardiac position, rotation, or tilting may modify the synchrony of regional wall motion and myocardial fiber shortening; reduced intrathoracic space may limit ventricular filling and modify preload and regional wall stress; and mechanical constraint may contribute to regional intraventricular dyssynchrony [35,36]. In addition, abnormal thoracic anatomy may differentially affect echocardiographic beam orientation and myocardial tracking throughout the cardiac cycle, introducing a methodological component to the measured reduction in strain. Indeed, previous observations in otherwise healthy subjects with PE suggested that reduced strain may reflect both mechanically induced intraventricular dyssynchrony and technical limitations related to chest wall abnormality rather than true subclinical myocardial dysfunction [37,38]. Thus, the term extrinsically mediated strain impairment may be more appropriate than “artifactual strain reduction”, as the observed abnormality may represent a combination of true mechanically constrained deformation and measurement-related effects.

This distinction has important implications for strain interpretation. An abnormal strain value describes altered myocardial deformation but does not, by itself, establish its biological origin. In the absence of structural heart disease, particularly when conventional systolic function is preserved and strain reduction preferentially involves basal myocardial segments, the possibility of an extracardiac mechanical contribution should therefore be considered before the finding is attributed to intrinsic myocardial dysfunction. Within this conceptual framework, thoracic geometry represents an additional determinant of myocardial strain that complements the traditional influences of loading conditions, ventricular geometry, and technical factors. The following sections examine the clinical models in which this interaction has been most consistently demonstrated.

## 3. Literature Search and Evidence Selection

A narrative literature search was conducted by A.S. using PubMed/MEDLINE, Scopus, and Embase from database inception to 10 August 2026; the searches were performed between 1 and 10 August 2026. No lower publication-date restriction was applied. The search aimed to identify relevant evidence on the potential influence of extracardiac mechanical determinants, particularly thoracic geometry, on myocardial deformation. The search strategy combined terms related to myocardial deformation with terms describing thoracic morphology, extracardiac mechanical determinants, and the clinical models considered in this review. Search terms included “myocardial strain”, “global longitudinal strain”, “speckle-tracking echocardiography”, “thoracic geometry”, “antero-posterior thoracic diameter”, “modified Haller index”, “pectus excavatum”, “mitral valve prolapse”, “pregnancy”, “obesity”, “relative apical sparing”, and “artifactual strain changes”. These terms were used in different combinations according to the database and topic being explored. A.S. screened the retrieved records for relevance based on titles and abstracts and subsequently evaluated potentially pertinent full-text articles for inclusion in the narrative synthesis. Reference lists of relevant articles were also examined to identify additional pertinent publications. Original studies, systematic reviews, meta-analyses, consensus documents, and clinically relevant reviews addressing myocardial deformation, thoracic morphology, or potential extracardiac mechanical determinants of strain were considered. Particular attention was given to studies reporting global and/or regional myocardial strain in relation to thoracic conformation or conditions potentially modifying the mechanical environment surrounding the heart. Articles were selected on the basis of their relevance to the mechanistic framework and clinical objectives of the present review. No formal systematic-review protocol, predefined quantitative synthesis, or risk-of-bias assessment was performed, consistent with the narrative nature of the article.

## 4. Pectus Excavatum: A Prototypical Model of Extrinsically Mediated Myocardial Strain Impairment

PE represents the most intuitive clinical model for investigating the influence of thoracic geometry on myocardial deformation. The characteristic posterior displacement of the sternum reduces the A–P thoracic diameter and the retrosternal space available for the heart, potentially exposing the cardiac chambers to chronic external mechanical constraint [39,40]. Although abnormalities in cardiac function have been documented in individuals with PE by both transthoracic echocardiography (TTE) [41,42,43,44] and cardiac magnetic resonance (CMR) studies [45,46,47,48], distinguishing true myocardial dysfunction from the mechanical consequences of altered chest geometry remains challenging. In this setting, myocardial strain imaging provides particularly interesting information because reduced deformation may occur despite preserved conventional indices of ventricular systolic function.

The right ventricle (RV) may be particularly susceptible to the mechanical consequences of PE because of its anterior position immediately behind the sternum. Posterior sternal displacement may alter RV geometry through direct compression and distortion, with reported changes including narrowing of the RV outflow tract, modification of RV cavity shape, and regional deformation of the RV wall [38]. These geometric alterations may impair RV diastolic filling, reduce effective preload and stroke volume, and modify regional RV systolic mechanics [38]. Importantly, the mechanical consequences of PE are unlikely to remain confined to the right ventricle. Because the two ventricles are mechanically coupled through the shared interventricular septum and enclosed within the same pericardial space, changes in RV loading, shape, and filling may influence LV geometry and motion through ventricular interdependence. Abnormal or paradoxical septal motion, including a “septal bounce”, has indeed been described in PE and may further modify regional LV deformation patterns [38]. Thus, altered strain measurements in PE may reflect not only direct external constraint on individual myocardial regions, but also secondary changes in biventricular interaction, septal mechanics, and loading conditions. These mechanisms should be considered when interpreting both RV and LV strain abnormalities in individuals with restrictive thoracic geometry.

In a study of otherwise healthy adults with PE, defined by an MHI > 2.5, both right- and left-sided cardiac chamber dimensions and stroke volume were significantly reduced compared with matched controls, whereas LVEF and conventional indices of diastolic function were preserved [31]. Despite the absence of conventional evidence of systolic dysfunction, myocardial deformation was markedly reduced, involving LV longitudinal, circumferential, and radial strain as well as right ventricular free-wall (RVFW) longitudinal strain. Importantly, the magnitude of these abnormalities was closely related to the severity of thoracic deformity: LV-GLS, global circumferential strain, global radial strain, and RVFW strain were all significantly correlated with MHI in subjects with PE but not in controls. These findings suggested that increasing thoracic constraint progressively modifies myocardial deformation and raised the possibility that reduced strain in PE may reflect mechanically induced alterations in ventricular motion and intraventricular synchrony rather than primary myocardial dysfunction.

A representative example illustrating the integrated assessment of thoracic morphology and myocardial deformation in an individual with PE is shown in Figure 3**,** combining MHI assessment with STE and demonstrating reduced LV-GLS predominantly driven by impaired basal deformation.

The regional distribution of strain abnormalities provides further support for this mechanical interpretation. External constraint imposed by a reduced sterno-vertebral distance does not appear to affect the ventricle uniformly, with deformation abnormalities being particularly evident in the basal and mid-ventricular regions. This spatial heterogeneity is relevant because the basal myocardium is likely to be more directly influenced by the restricted intrathoracic space, whereas the apex retains comparatively greater freedom of motion. Consequently, PE may generate a characteristic base-to-apex deformation gradient in which basal strain is disproportionately reduced relative to apical strain. Importantly, despite the overall reduction in myocardial deformation, the physiological base-to-apex gradient, characterized by progressively greater deformation toward the apex [49,50,51], appears to remain preserved in individuals with PE [31,32]. This preservation of the normal regional deformation hierarchy, particularly when accompanied by preserved conventional systolic function, argues against diffuse intrinsic myocardial dysfunction and instead supports a mechanically mediated alteration of myocardial deformation, whereby regional strain abnormalities may reflect the anatomical distribution of external mechanical forces rather than regional myocardial injury.

Particularly compelling evidence comes from the evaluation of PE during early life. In infants with PE, reduced cardiac chamber dimensions and impaired biventricular deformation were already detectable during the perinatal period and persisted at approximately 40 days of life [32]. During postnatal growth, the increase in A–P thoracic diameter was significantly smaller in PE infants than in controls, thereby maintaining the anatomical conditions for external cardiac compression. A narrow A–P diameter was identified as the major anatomical determinant associated with smaller cardiac chambers and reduced myocardial deformation, with strain impairment involving all three principal directions and being particularly evident at the mid and basal levels. Moreover, a strong inverse relationship between MHI and myocardial deformation indices was observed in PE infants, but not in controls, both during the perinatal period and at follow-up.

The neonatal model is particularly informative because it minimizes the potential influence of acquired cardiovascular disease and long-standing exposure to conventional cardiovascular risk factors. In these clinically healthy infants, the coexistence of preserved conventional biventricular contractile function with abnormalities in multidirectional strain, together with the close relationship between strain impairment and the degree of chest wall deformity, argues against intrinsic myocardial dysfunction as the sole explanation. Furthermore, increased LV apical rotation observed in PE infants has been proposed as a compensatory response aimed at maintaining ventricular performance in the presence of basal mechanical compression [32].

Taken together, findings obtained across adult and neonatal populations support PE as a prototypical model of extrinsically mediated myocardial strain impairment. They also suggest that the A–P thoracic diameter may be more than a simple descriptor of chest morphology, representing a clinically relevant mechanical determinant of measured myocardial deformation. Accordingly, reduced global or regional strain in otherwise healthy individuals with PE should not automatically be interpreted as evidence of subclinical cardiomyopathy. Instead, the severity of thoracic restriction and its regional mechanical consequences should be incorporated into the interpretation of strain findings, particularly when conventional ventricular function is preserved and deformation abnormalities predominantly involve the basal myocardial segments.

## 5. Mitral Valve Prolapse: Interplay Between Intrinsic and Extrinsic Determinants of Regional Myocardial Deformation

Mitral valve prolapse (MVP) represents a particularly intriguing model in which intrinsic cardiac mechanisms and extrinsic mechanical factors may coexist in determining abnormalities of myocardial deformation. Speckle-tracking echocardiographic studies have reported heterogeneous findings in individuals with MVP, ranging from preserved or enhanced global deformation to reduced LV longitudinal strain [52,53,54,55,56,57,58,59,60,61,62,63,64,65]. Importantly, however, the most consistent abnormality appears to be regional rather than global, with preferential involvement of the basal inferolateral myocardium.

This concept was recently supported by a systematic review and meta-analysis including 15 studies, 1088 individuals with MVP, and 591 healthy controls [66]. Overall, LV-GLS magnitude was modestly but significantly lower in individuals with MVP than in controls (19.4 ± 3.4% vs. 21.1 ± 2.8%), corresponding to a small-to-moderate pooled effect. More importantly, regional strain analysis consistently identified a preferential reduction in deformation at the LV basal inferolateral segments across longitudinal, circumferential, and radial directions, accompanied by relative preservation of apical deformation. Thus, the myocardial mechanical phenotype associated with MVP appears to be characterized less by homogeneous global dysfunction than by a distinctive regional base-to-apex deformation gradient.

Several intrinsic mechanisms may contribute to this regional pattern. The close anatomical relationship between the mitral apparatus and the basal inferolateral myocardium may expose these segments to abnormal mechanical forces generated by systolic leaflet motion, annular dynamics, and traction transmitted through the papillary muscles and chordal apparatus [67,68,69]. Such mechanisms are particularly relevant in more complex MVP phenotypes and may coexist with myocardial remodeling. However, intrinsic myocardial abnormalities may not fully account for reduced basal strain in all individuals with MVP, particularly in otherwise healthy subjects without significant mitral regurgitation or other evidence of structural myocardial disease.

Thoracic morphology provides a potentially complementary explanation. Importantly, MVP is frequently associated with anterior chest wall and thoracic skeletal abnormalities, which may result in a flat or concave chest configuration and a reduced A–P thoracic diameter, even in the absence of severe chest wall deformity [70,71,72,73,74]. Such thoracic narrowing may impose an additional mechanical constraint on the heart, potentially accentuating the preferential impairment of basal myocardial deformation. In a previous echocardiographic investigation, individuals with MVP and a narrow chest configuration showed more pronounced abnormalities in LV deformation than those with less restrictive thoracic geometry [61]. In particular, increasing thoracic restriction was associated with preferential attenuation of basal myocardial deformation, suggesting that extracardiac mechanical constraint may modulate the regional strain phenotype observed in MVP. This concept is illustrated in Figure 4, which integrates anthropometric assessment of thoracic geometry with conventional echocardiographic identification of MVP and the corresponding LV longitudinal strain pattern. The representative individual exhibits a narrow thoracic configuration associated with echocardiographic MVP and reduced LV-GLS, predominantly driven by impaired basal longitudinal deformation with relative preservation of mid-ventricular and apical strain. This regional pattern supports the concept that the myocardial mechanical phenotype of MVP may result from the interplay between forces generated by the mitral apparatus and extrinsic constraints imposed by thoracic geometry, rather than necessarily reflecting diffuse intrinsic myocardial dysfunction.

A plausible interpretation is therefore that the regional strain pattern observed in MVP may result from the interaction of intrinsic valvular–ventricular forces and extrinsic thoracic mechanical constraints, rather than from a single mechanism. A narrow A–P thoracic diameter may further restrict the motion of basal myocardial segments already exposed to abnormal forces generated by the prolapsing mitral apparatus [75,76], whereas the apical myocardium remains relatively unconstrained. This concept may be regarded as a potential “double-hit” mechanical model, in which valvular–ventricular interaction and thoracic geometry act synergistically to accentuate basal deformation abnormalities. Importantly, this hypothesis should not be interpreted as an alternative to established myocardial mechanisms in MVP, but rather as an additional factor potentially contributing to the heterogeneity of strain findings among otherwise similar individuals.

The preferential basal involvement also provides an important link between MVP and the relative apical sparing phenotype. The combination of reduced basal deformation and comparatively preserved apical strain can generate a base-to-apex gradient resembling relative apical sparing, despite the absence of an infiltrative cardiomyopathy. Evidence from the broader analysis of relative apical sparing supports the possibility that reduced A–P thoracic dimensions may mechanically constrain basal LV segments while leaving the apex relatively free to deform. Thoracic geometry may therefore represent one of the factors contributing to an extrinsic mechanical phenocopy of apical sparing in selected individuals with MVP.

From a clinical perspective, these observations caution against automatically interpreting reduced basal strain in MVP as evidence of intrinsic myocardial injury. The significance of an abnormal strain pattern should instead be evaluated in the context of mitral regurgitation severity, ventricular morphology and function, the overall MVP phenotype, and thoracic conformation. Assessment of the A–P thoracic diameter and, when appropriate, the MHI may provide additional information for distinguishing a predominantly extrinsic mechanical contribution from myocardial abnormalities warranting further investigation.

## 6. Healthy Pregnancy: A Dynamic and Reversible Model of Extrinsically Mediated Strain Impairment

Pregnancy represents a unique physiological model for investigating the relationship between extracardiac mechanical conditions and myocardial deformation. During normal gestation, the cardiovascular system undergoes profound hemodynamic adaptations, including increases in circulating blood volume, cardiac output, and heart rate, together with changes in systemic vascular resistance and ventricular loading conditions [77,78,79]. These physiological modifications have traditionally been considered the main determinants of changes in myocardial mechanics during pregnancy. However, the progressive enlargement of the uterus and upward displacement of the diaphragm also modify the spatial relationship between the heart and surrounding thoracic structures [80,81,82], suggesting that mechanical changes in the available intrathoracic space may contribute to the variations in myocardial strain documented across several studies using speckle-tracking echocardiography (STE) in healthy pregnant women [83,84,85,86,87,88,89,90].

This hypothesis was investigated longitudinally in 50 healthy pregnant women who underwent echocardiographic and thoracic morphometric assessment during the first trimester (12–14 weeks), third trimester (36–38 weeks), and 6–9 weeks after delivery [90]. Although LVEF remained substantially unchanged throughout pregnancy, myocardial strain parameters showed a small but significant reduction during gestation followed by recovery after delivery. Importantly, stratification according to thoracic conformation revealed that this pattern was not uniformly observed across the study population. Women with a concave-shaped chest wall, defined by an MHI > 2.5, showed a progressive reduction in myocardial strain parameters during pregnancy, whereas no comparable deterioration was observed in women with an MHI ≤ 2.5. In the third trimester, MHI was strongly correlated with both LV-GLS and LV global circumferential strain. A representative example of this phenotype is shown in Figure 5, illustrating a healthy woman in the third trimester of pregnancy with a narrow A–P thoracic diameter and concomitant reduction in LV-GLS, predominantly driven by impaired longitudinal deformation of the basal myocardial segments with relative preservation of mid-ventricular and apical strain. The integrated assessment of thoracic morphometry and regional myocardial deformation illustrates how a restrictive thoracic configuration may contribute to the characteristic base-to-apex strain gradient observed during late pregnancy.

The longitudinal nature of these observations is particularly informative. If the reduction in myocardial strain during uncomplicated pregnancy were predominantly attributable to intrinsic myocardial dysfunction, its close dependence on thoracic conformation and subsequent postpartum normalization would be difficult to explain. Conversely, the findings are consistent with a dynamic mechanical model in which progressive diaphragmatic elevation during pregnancy further reduces the space available to the heart, particularly in women with a pre-existing narrow A–P thoracic diameter. The resulting external constraint may transiently alter cardiac position and spatial orientation, ventricular geometry, myocardial excursion, and regional deformation without necessarily indicating structural myocardial injury. The disappearance of these abnormalities after delivery, when the mechanical effects of the gravid uterus and diaphragmatic elevation are relieved, provides additional support for their reversible and predominantly mechanical nature.

The influence of thoracic geometry during pregnancy appears to extend beyond myocardial strain alone. Previous investigations have shown that anterior chest wall conformation may also influence stroke volume and ventricular–arterial coupling during normal pregnancy [91], reinforcing the concept that cardiovascular adaptation to gestation occurs within an anatomical environment whose dimensions may substantially modulate cardiac mechanics. In particular, women with a smaller body surface area and a narrower A–P thoracic diameter, typically resulting in a higher MHI, exhibit smaller cardiac chamber dimensions, lower preload and stroke volume, and a different VAC response to pregnancy compared with women with a normal chest conformation. In these women, the physiological volume expansion of pregnancy may be less effectively accommodated within the restricted thoracic space, resulting in a less pronounced increase in stroke volume and a progressive increase in VAC [91]. Thus, cardiovascular adaptation to gestation occurs within an anatomical environment whose dimensions may substantially modulate cardiac mechanics. Accordingly, two women exposed to broadly similar physiological hemodynamic changes during pregnancy may exhibit different myocardial deformation responses depending, at least in part, on their body size and thoracic geometry.

Healthy pregnancy can therefore be considered a dynamic and potentially reversible model of extrinsically mediated myocardial strain impairment. Unlike PE, in which the mechanical constraint is primarily related to a relatively fixed chest wall abnormality, pregnancy introduces a transient extracardiac force superimposed on the pre-existing thoracic anatomy. This distinction is clinically relevant because modest reductions in myocardial strain during uncomplicated late pregnancy, particularly in women with preserved LVEF and no other evidence of cardiac disease, should not automatically be interpreted as subclinical myocardial dysfunction. Assessment of thoracic conformation and, particularly, the A–P thoracic diameter may help distinguish physiological or mechanically mediated changes in myocardial deformation from findings that warrant investigation for an underlying pregnancy-related cardiac disorder.

## 7. Obesity and Body Fat Distribution: The Interaction Between Abdominal Adiposity and Thoracic Geometry

Obesity is commonly associated with alterations in cardiac structure and function [92,93], and reduced myocardial strain has been reported even in individuals without clinically overt cardiovascular disease [94,95,96,97,98,99]. Such abnormalities are generally interpreted as evidence of early obesity-related myocardial dysfunction, potentially mediated by metabolic abnormalities, systemic inflammation, insulin resistance, neurohormonal activation, and myocardial lipid accumulation [100,101,102,103,104,105,106]. However, obesity also profoundly modifies body geometry and the mechanical environment surrounding the heart. Accordingly, at least part of the myocardial strain impairment observed in otherwise healthy individuals with obesity may reflect extrinsic mechanical influences rather than exclusively intrinsic myocardial abnormalities.

The potential contribution of body geometry was specifically investigated by combining thoracic morphometry with conventional and STE in subjects with obesity [107]. In this population, waist circumference and the non-radiological A–P thoracic diameter were the only variables independently associated with impaired LV-GLS, whereas body mass index (BMI), conventional cardiovascular risk factors, biochemical variables, LV mass index, LVEF, and other conventional echocardiographic parameters were not significantly associated with reduced LV-GLS. A waist circumference ≥ 100.5 cm and an A–P thoracic diameter ≤ 17 cm represented the best cut-off values for identifying impaired LV-GLS within the population investigated. These findings suggest that the mechanical consequences of obesity may depend less on overall body mass than on the spatial distribution of adipose tissue and the anatomical space available to the heart. This phenotype is illustrated in Figure 6, showing a healthy individual with obesity undergoing thoracic morphometric assessment, in whom a relatively narrow A–P thoracic diameter is associated with reduced LV-GLS, predominantly driven by impaired longitudinal deformation of the basal myocardial segments, with relative preservation of mid-ventricular and apical strain.

A plausible mechanical explanation involves the combined effects of abdominal adiposity and a relatively narrow A–P thoracic dimension. Increased abdominal volume may promote upward displacement of the diaphragm [108], while a reduced sterno-vertebral distance limits the available intrathoracic space. Acting simultaneously from inferior and anterior directions, these factors may modify cardiac position, chamber geometry, and myocardial excursion. Consistent with this hypothesis, thoracic and abdominal anthropometric measurements showed stronger relationships with LV-GLS than conventional clinical and echocardiographic variables. Even in the presence of a relatively circular transverse chest configuration, a shorter A–P thoracic diameter was associated with impaired LV deformation, supporting the concept that the absolute or relative restriction of the A–P dimension may be more relevant than the external appearance of the chest alone.

Further support for this concept derives from the comparison of different patterns of fat distribution [109]. In healthy women with obesity, myocardial deformation differed substantially between android (“apple-shaped”) and gynoid (“pear-shaped”) phenotypes. Reduced LV-GLS was considerably more frequent among women with android obesity, being observed in 65% of this group compared with only 5% of women with gynoid obesity. Moreover, LV-GLS showed strong relationships with waist-to-hip ratio (WHR), MHI, and epicardial adipose tissue (EAT), suggesting that abdominal, thoracic, and intrathoracic adiposity may act synergistically in modulating myocardial deformation. By contrast, the greater myocardial deformation observed in women with gynoid obesity was associated with a more spheroidal transverse thoracic configuration, potentially resulting in less external cardiac constraint and displacement.

Importantly, thoracic geometry and fat distribution are unlikely to account for the full spectrum of myocardial deformation abnormalities observed in obesity. Recent evidence in young adults with overweight or obesity identified a distinct phenotype characterized by impaired three-dimensional global circumferential strain despite preserved longitudinal strain, suggesting preferential involvement of mid-wall rather than subendocardial mechanics [110]. In that study, the skeletal muscle mass-to-fat (SMM-to-Fat) ratio showed a stronger relationship with circumferential strain than skeletal muscle mass alone and remained independently associated with circumferential deformation after adjustment for age and C-peptide. These observations highlight body composition, and particularly the balance between metabolically active skeletal muscle and adipose tissue, as another potentially relevant determinant of myocardial mechanics in obesity. Accordingly, the relative contribution of thoracic constraint, adipose distribution, body composition, metabolic factors, and loading conditions may differ substantially among individuals and may also result in distinct strain phenotypes. Thoracic morphometry should therefore be considered complementary to, rather than a substitute for, a broader assessment of body composition and cardiometabolic phenotype in obesity.

Taken together, these findings challenge the use of BMI as the principal anthropometric variable when interpreting myocardial strain in obesity and suggest that both body composition and body geometry should be considered. Individuals with similar BMI may have markedly different distributions of adipose tissue and thoracic geometries and, consequently, different mechanical effects on the heart [111,112]. In this context, android obesity may represent a mechanically less favorable phenotype than gynoid obesity, because central adiposity may interact with a narrow A–P thoracic dimension to restrict the space available for normal cardiac motion. Interestingly, even among healthy non-obese controls, mildly reduced LV-GLS was observed in a subgroup characterized by an A–P thoracic diameter < 13.5 cm, further suggesting that thoracic restriction itself may influence strain independently of obesity.

Obesity therefore represents a more complex model of extrinsically mediated myocardial strain impairment than PE or pregnancy. Rather than a single anatomical constraint, multiple and potentially interacting determinants—including body composition and muscle-to-fat balance, metabolic and inflammatory pathways, abdominal adiposity, thoracic conformation, EAT, diaphragmatic position, and cardiac displacement—may influence measured myocardial deformation. The relative contribution of these mechanisms is likely to vary among individuals, particularly according to obesity severity, duration, body composition, and associated comorbidities [113,114]. Accordingly, reduced strain in an otherwise healthy individual with obesity should not automatically be attributed to subclinical myocardial disease without considering the accompanying body composition, cardiometabolic, anthropometric, and thoraco-abdominal mechanical phenotype. In this setting, assessment of body composition, together with simple anthropometric and thoracic measurements such as waist circumference and A–P thoracic diameter, may provide complementary contextual information for interpreting abnormal myocardial strain.

## 8. From Direct Mechanical Constraint to Multifactorial Models of Myocardial Deformation

The principal studies [31,32,61,90,107] specifically examining the relationship between thoracic morphology and myocardial deformation across the clinical models discussed in this review are summarized in Table 1.

Collectively, they show a recurring association between thoracic morphometric parameters, particularly MHI and A–P thoracic diameter, and global or regional myocardial strain abnormalities across markedly different clinical settings. However, the available evidence remains preliminary. Most studies were observational, single-center investigations involving relatively small and selected populations, and several were conducted by the same research group using similar acquisition protocols and the same echocardiographic platform. Moreover, the investigated populations, thoracic phenotypes, strain components, and potential competing mechanisms differed substantially across studies. These methodological characteristics limit external generalizability and preclude causal inference. Therefore, the morphometric values emerging from these cohorts should be considered exploratory markers rather than validated diagnostic thresholds. Independent multicenter studies using different ultrasound vendors, standardized thoracic acquisition protocols, larger and more heterogeneous populations, and, where appropriate, multimodality imaging are required to determine the reproducibility and incremental clinical value of this proposed framework.

Taken together, these clinical models should not be regarded as mechanistically equivalent. PE represents the most direct anatomical model of external cardiac constraint, in which reduction in the retrosternal space provides an intuitive link between thoracic geometry and altered cardiac mechanics [31,38]. By contrast, MVP, pregnancy, and obesity represent more complex biological settings in which thoracic geometry constitutes only one of several potential determinants of myocardial deformation. In MVP, intrinsic valvular–ventricular forces and myocardial remodeling may coexist with extracardiac thoracic constraint [63,67,68,69]; during pregnancy, major physiological changes in preload, cardiac output, heart rate, systemic vascular resistance, and ventricular loading occur simultaneously with progressive diaphragmatic elevation and changes in intrathoracic geometry [77,78,79,80,81,82,83,84,85,86,87,88,89,90]; and in obesity, metabolic, inflammatory, insulin-resistant, neurohormonal, and myocardial mechanisms may accompany the mechanical effects of abdominal adiposity and altered thoracic conformation [92,93,94,95,96,97,98,99,100,101,102,103,104,105,106,107]. Accordingly, the association between thoracic geometry and myocardial strain in these latter conditions should not be interpreted as evidence that extracardiac constraint is the predominant or exclusive mechanism. Rather, thoracic morphology should be considered one component of a multifactorial framework in which intrinsic myocardial, hemodynamic, metabolic, and extracardiac mechanical influences may coexist and interact.

A further methodological consideration applies across all these clinical models. Thoracic conformation and patient position may alter the spatial position, rotation, and angulation of the heart within the chest, thereby affecting acoustic windows, ultrasound beam orientation, image quality, and myocardial tracking. Current two-dimensional speckle-tracking algorithms may be particularly sensitive to out-of-plane motion and dynamic changes in beam–wall angulation throughout the cardiac cycle; consequently, some of the observed strain abnormalities may reflect geometry-dependent measurement effects in addition to genuine changes in myocardial mechanics [13,38]. Importantly, reliable strain analysis may be particularly challenging in individuals with suboptimal acoustic windows, in whom inadequate endocardial definition or incomplete visualization of myocardial segments may compromise tracking quality and the reliability of both global and regional strain measurements. This limitation may be especially relevant in patients with marked chest wall deformity or obesity and should be carefully considered before attributing an abnormal strain value to either intrinsic myocardial disease or an extrinsic mechanical mechanism. When image quality does not permit reliable tracking, strain measurements should therefore be interpreted with particular caution or considered technically inadequate rather than assigned biological significance. This potential interaction between anatomical, mechanical, and technical determinants further reinforces the need for cautious interpretation of strain abnormalities in individuals with altered thoracic geometry.

## 9. Relative Apical Sparing Beyond Cardiac Amyloidosis: An Extrinsic Mechanical Phenocopy

Relative apical sparing (RASP) is a distinctive pattern of LV longitudinal deformation characterized by a disproportionate reduction in basal and mid-ventricular strain with relative preservation of apical deformation [115,116,117]. Although classically associated with cardiac amyloidosis, RASP is not disease-specific and may occur in several other cardiac conditions [118,119,120,121,122,123,124,125,126,127]. Its presence should therefore be regarded as a deformation phenotype rather than as a direct surrogate of a specific myocardial substrate. Importantly, a base-to-apex strain gradient may result not only from intrinsic myocardial disease but also from regional differences in loading, ventricular geometry, fiber architecture, and extracardiac mechanical constraints.

Thoracic geometry provides a plausible extracardiac mechanism for an apical-sparing-like pattern. A reduced A–P thoracic dimension may be associated with preferential restriction of basal and mid-ventricular motion while leaving the apex relatively less restricted, thereby potentially contributing to a base-to-apex deformation gradient in the absence of intrinsic myocardial disease. As discussed above, observations in PE and MVP support this concept, as both conditions may exhibit preferential impairment of basal or mid-ventricular deformation with relative preservation of apical mechanics [31,32,66]. In MVP, the higher prevalence of associated anterior chest wall abnormalities [70,71,72,73,74] further supports a potential contribution of extracardiac mechanical constraint to this regional phenotype. Thus, relative apical preservation may, at least in selected individuals, represent the mechanical counterpart of preferential restriction of more basal ventricular regions.

These considerations are particularly relevant to the interpretation of the characteristic strain “bull’s-eye”. A visually striking apical-sparing pattern may suggest cardiac amyloidosis, but a similar geometric appearance may arise from different pathophysiological mechanisms. RASP should therefore be interpreted within the broader clinical and imaging context rather than in isolation. Moreover, differences in RASP calculation, segmental averaging, myocardial layer selection, tracking algorithms, temporal smoothing, and the use of absolute versus signed strain values may influence its magnitude and limit the generalizability of fixed diagnostic thresholds [128].

Recognition of a potential extrinsic mechanical phenocopy of RASP may have relevant diagnostic implications. In an otherwise healthy individual with preserved LVEF, no unexplained LV hypertrophy, and no additional clinical or imaging evidence of infiltrative cardiomyopathy, preferential basal strain impairment should prompt consideration of thoracic geometry before attributing the strain pattern to a specific myocardial disease. Assessment of thoracic morphology, including the A–P thoracic diameter, may therefore provide useful complementary information when an otherwise unexplained apical-sparing pattern is encountered. Importantly, the identification of a restrictive thoracic configuration should never be used to dismiss or exclude an infiltrative or other intrinsic myocardial disease. When clinical, electrocardiographic, biomarker, morphological, or CMR findings raise suspicion of an underlying cardiomyopathy, appropriate diagnostic investigation remains warranted irrespective of thoracic morphology. Intrinsic myocardial disease and extrinsic mechanical influences may also coexist, and thoracic morphometry should therefore be regarded only as complementary contextual information, rather than as an alternative diagnostic explanation.

Accordingly, RASP illustrates a broader principle underlying this review: the spatial distribution of myocardial strain abnormalities does not necessarily identify their biological origin. Similar deformation patterns may arise from intrinsic myocardial pathology or from altered mechanical boundary conditions surrounding an otherwise structurally normal heart. Recognizing this distinction may reduce diagnostic misclassification and reinforces the need to integrate myocardial strain with conventional echocardiography, clinical pre-test probability, ECG and biomarker findings, multimodality imaging when clinically indicated, and, when appropriate, thoracic morphometric assessment.

## 10. Other Thoracic and Physiological Models Potentially Influencing Myocardial Deformation

The potential influence of the extracardiac mechanical environment on myocardial deformation is unlikely to be restricted to PE, MVP, pregnancy, or obesity. Other anatomical and physiological conditions that modify thoracic geometry, cardiac position, loading conditions, or cardiopulmonary interactions may provide complementary models for understanding variations in myocardial strain.

Scoliosis represents another relevant model in which thoracic geometry may influence cardiac structure and myocardial mechanics. An increased prevalence of MVP has been reported in individuals with scoliosis, supporting an association between spinal/thoracic morphology and cardiac phenotype [70]. Importantly, Li et al. demonstrated by 2D-STE significantly reduced LV longitudinal, radial, and circumferential strain in children and adolescents with idiopathic scoliosis compared with healthy controls [129]. Moreover, several deformation parameters correlated with the Cobb angle, suggesting a relationship between scoliosis severity and altered LV mechanics [129]. These findings support scoliosis as an additional model in which altered thoracic geometry and cardiopulmonary interactions may contribute to myocardial deformation abnormalities.

Straight back syndrome (SBS) represents an even more direct anatomical model. Loss of normal thoracic kyphosis reduces the A–P thoracic diameter and may restrict the heart between the sternum and vertebral column. An increased prevalence of MVP has been reported in individuals with SBS, further supporting an association between thoracic morphology and cardiac phenotype [72]. The concomitant occurrence of MVP has also been proposed to reflect compression-induced mitral valve deformation secondary to A–P thoracic narrowing [72]. Although dedicated STE studies are lacking, SBS provides a relevant model in which reduced thoracic dimensions may potentially influence myocardial mechanics and strain measurements.

A different mechanical paradigm is provided by chronic obstructive pulmonary disease (COPD). Pulmonary hyperinflation may reduce cardiac preload, alter cardiac chamber geometry, and increase intrathoracic pressure, potentially compressing both right- and left-sided cardiac chambers. Independent STE studies have demonstrated impaired biventricular deformation even in COPD patients without advanced pulmonary hypertension; for example, Sabit et al. reported subclinical LV and RV dysfunction despite relatively preserved conventional systolic indices [130]. Thus, in COPD, reduced strain may reflect a complex interaction among altered loading conditions, pulmonary vascular disease, ventricular interdependence, hypoxemia, systemic inflammation, and lung hyperinflation rather than a purely intrinsic myocardial process [131].

Finally, the athlete’s heart illustrates how myocardial deformation may vary substantially in response to physiological loading without necessarily indicating disease. Prolonged endurance exercise can induce transient reductions in LV strain, whereas chronic training is generally associated with preserved physiological remodeling. Importantly, loading conditions and body position can themselves influence deformation measurements. Kandels et al. demonstrated a marked reduction in global LV deformation in athletes examined in the upright position, with predominant involvement of the basal inferior and posterolateral segments [132].

These observations are particularly relevant to the present framework because they demonstrate that regional strain patterns may change substantially in structurally normal hearts as a function of physiological and mechanical boundary conditions. Collectively, scoliosis, SBS, COPD, and the athlete’s heart broaden the spectrum of conditions in which myocardial strain should be interpreted within its anatomical, loading, and mechanical context. However, unlike PE, direct evidence relating thoracic morphometry to myocardial deformation remains limited or absent in these settings, highlighting an important area for future investigation.

## 11. Thoracic Morphometry in Clinical Practice: The Role of the Modified Haller Index and A–P Thoracic Diameter

Recognition of a potential extrinsic contribution to myocardial strain impairment requires a simple and clinically applicable method for characterizing the mechanical environment surrounding the heart. Although visual inspection may identify obvious chest wall abnormalities, less pronounced variations in thoracic geometry can easily remain unrecognized during routine cardiovascular assessment. Quantitative thoracic morphometry may therefore provide useful complementary information, particularly when reduced global or regional myocardial strain is unexpectedly detected in an individual without other evidence of structural heart disease.

The Haller index (HI), originally developed for the assessment of PE, is conventionally calculated as the ratio between the transverse thoracic diameter and the shortest A–P distance between the sternum and vertebral column [133]. However, conventional HI assessment requires radiological imaging and is therefore poorly suited to routine echocardiographic evaluation or repeated measurements. To overcome these limitations, the MHI was developed as a simple, non-radiological method combining an external measurement of the maximum L–L thoracic diameter with an echocardiographic measurement of the internal A–P diameter. The latter is obtained from the conventional parasternal long-axis view as the distance between the ultrasound entry point at the skin surface and the posterior wall of the descending thoracic aorta, which serves as an echocardiographic landmark approximating the anterior surface of the vertebral column [23]. The MHI is subsequently calculated as the L–L/A–P ratio.

This approach has several practical advantages. It is inexpensive, radiation-free, repeatable, and can be performed at the bedside using readily identifiable anatomical landmarks. Moreover, the methodology has been clinically validated against conventional radiological assessment, supporting its feasibility as an alternative method for quantitative characterization of thoracic conformation. Importantly, subsequent applications of the MHI have extended its potential relevance beyond the anatomical assessment of PE, showing associations between thoracic conformation and cardiac chamber geometry, ventricular filling, myocardial deformation, ventricular–arterial coupling, and other functional cardiovascular parameters [134]. These associations, however, should not be interpreted as establishing a direct causal effect of thoracic geometry on myocardial mechanics.

For the specific purpose of myocardial strain interpretation, however, calculation of the complete MHI may not always be necessary. The A–P thoracic diameter itself may represent the most immediate and clinically accessible morphometric parameter, because it provides an estimate of the sterno-vertebral space available to the heart and can be obtained from a standard echocardiographic view without additional radiological imaging or dedicated equipment. This concept is supported by observations showing that a reduced A–P thoracic diameter is closely associated with impaired myocardial deformation across different clinical models. In subjects with obesity, for example, the non-radiological A–P thoracic diameter, together with waist circumference, was independently associated with impaired LV-GLS, whereas several conventional clinical and echocardiographic variables were not [107]. Similarly, a narrow A–P diameter has emerged as an anatomical correlate of external cardiac constraint in subjects with PE and other restrictive thoracic phenotypes [38]. Importantly, these predominantly observational findings cannot establish whether the association with reduced strain reflects genuine mechanical restriction of myocardial motion, geometry-related effects on image acquisition and speckle tracking, or a combination of both mechanisms.

From a practical perspective, thoracic morphometry could therefore be incorporated as a contextual step in the interpretation of unexpected strain abnormalities, rather than as a universal screening procedure. When reduced LV-GLS, disproportionate basal strain impairment, or relative apical preservation is observed despite preserved conventional ventricular function, the echocardiographer should first review the clinical and imaging context and recognized determinants of strain variability, including loading conditions, image quality, frame rate, operator-related factors, and vendor-specific acquisition and post-processing algorithms, and consider whether thoracic geometry could be associated with or contribute to the observed pattern. Inspection of the chest wall and measurement of the A–P thoracic diameter from the parasternal long-axis view could represent a rapid first-line approach. If a restrictive thoracic configuration is suspected, complete MHI assessment may provide a more comprehensive quantitative characterization.

Across several investigations, an A–P thoracic diameter < 13.5 cm has consistently emerged as an exploratory morphometric marker associated with impaired LV-GLS, supporting a potential relationship between a particularly narrow thoracic configuration and altered myocardial deformation [31,32,61,90,91]. However, these observations derive predominantly from single-center studies conducted by the same research group and in selected populations, and should therefore be interpreted cautiously. Accordingly, the 13.5 cm value should not currently be regarded as a universal diagnostic threshold, but rather as a preliminary morphometric marker identifying a thoracic configuration in which an extrinsic mechanical contribution to strain impairment may be more likely. External validation in larger, independent, and more heterogeneous cohorts is required, together with the establishment of age-, sex-, and body-size-adjusted reference ranges and evaluation of whether indexed or normalized A–P measurements may provide greater diagnostic accuracy.

Thoracic morphometry should likewise not be considered a substitute for appropriate investigation when myocardial disease is clinically suspected. Rather, its potential value lies in providing a complementary framework for strain interpretation. A reduced A–P diameter or increased MHI does not prove that an abnormal strain value is mechanically mediated; instead, it identifies an anatomical setting in which an extrinsic contribution becomes more plausible. Conversely, normal thoracic geometry should prompt greater consideration of alternative hemodynamic, technical, or intrinsic myocardial mechanisms.

Accordingly, incorporation of simple thoracic measurements into routine echocardiographic reasoning may help shift strain interpretation from a purely numerical approach toward a more comprehensive mechanistic assessment of myocardial deformation. In selected individuals, adding a measurement that requires only a conventional parasternal long-axis image may provide additional mechanical context for an otherwise unexplained reduction in strain and help prevent the automatic equation of abnormal deformation with subclinical myocardial disease. At present, however, thoracic morphometry should be regarded as a hypothesis-generating contextual marker rather than evidence of a causal mechanism, pending prospective, externally validated, and preferably multimodality studies capable of disentangling genuine mechanical effects from measurement-related influences.

## 12. Clinical Implications: A Mechanistic Approach to Myocardial Strain Interpretation

The recognition that myocardial deformation may be influenced by extracardiac mechanical factors has important implications for strain interpretation. Although reduced LV-GLS with preserved LVEF is commonly regarded as a marker of subclinical myocardial dysfunction, this assumption should not be applied indiscriminately. In individuals without overt structural heart disease, abnormal strain may reflect the combined influence of myocardial properties, loading conditions, ventricular geometry, and extrinsic mechanical constraints.

The regional distribution of strain abnormalities may provide an important clue. Extrinsically mediated impairment appears to preferentially involve the basal and, to a lesser extent, mid-ventricular segments, with relative preservation of apical deformation. Thus, a reduced GLS predominantly driven by basal segments, particularly when disproportionate to otherwise preserved cardiac structure and function, should raise the possibility of an extrinsic mechanical contribution.

Thoracic morphology may therefore provide useful context. A narrow A–P thoracic diameter (<13.5 cm) may restrict the space available for cardiac motion and contribute to regional deformation abnormalities. When unexpectedly reduced strain is identified, image quality and recognized technical and hemodynamic determinants should first be reviewed, followed by assessment of the regional strain pattern. In the presence of predominant basal impairment with relative apical preservation, thoracic conformation can be assessed visually and by measuring the A–P diameter from the parasternal long-axis view; when appropriate, complete MHI assessment may provide additional quantitative characterization.

Figure 7 summarizes a practical stepwise approach integrating conventional echocardiographic findings, regional strain distribution, clinical red flags, and thoracic morphometry to guide the interpretation of unexpectedly reduced myocardial strain in individuals without overt structural heart disease.

Importantly, “abnormal strain” and “subclinical myocardial dysfunction” should not be considered interchangeable. Likewise, relative apical sparing should not automatically imply infiltrative cardiomyopathy, as thoracic constraint may represent a potential mechanical phenocopy. Conversely, a narrow A–P diameter (<13.5 cm) or increased MHI (>2.5) does not exclude myocardial disease, since intrinsic and extrinsic mechanisms may coexist. Thoracic morphometry should therefore be regarded as a contextual “mechanical marker” rather than a diagnostic endpoint.

When strain abnormalities coexist with structural cardiac abnormalities, significant valvular disease, abnormal diastolic function, elevated cardiac biomarkers, electrocardiographic abnormalities, arrhythmias, relevant symptoms, or other clinical red flags, further diagnostic evaluation remains warranted irrespective of thoracic morphology.

Overall, these observations support a shift from a purely numerical toward a multiparametric and mechanistic interpretation of myocardial strain, considering where deformation is reduced, whether other cardiac findings are concordantly abnormal, and the mechanical environment surrounding the heart. Such an approach may improve diagnostic specificity, reduce inappropriate classification of mechanically mediated strain abnormalities as subclinical myocardial disease, and better identify individuals requiring further diagnostic investigation.

## 13. Knowledge Gaps and Future Directions

Although available evidence supports an interaction between thoracic geometry and myocardial deformation, extrinsically mediated strain impairment remains insufficiently investigated. Most studies have involved small, selected populations, and several of the studies specifically addressing thoracic morphometry and myocardial strain were conducted by the same research group. Independent replication is currently limited, restricting the generalizability of current findings.

A major priority is the independent external validation of thoracic morphometric reference values. Although an MHI > 2.5 and an A–P thoracic diameter < 13.5 cm have emerged as exploratory markers of a restrictive thoracic configuration associated with impaired myocardial deformation, these values derive predominantly from selected cohorts and single-center experience and should not currently be considered diagnostic cut-offs. Large multicenter studies conducted by independent research groups are needed to establish age-, sex-, and body-size-adjusted reference values and determine whether absolute, indexed, or ratio-based measurements provide the most informative assessment.

Another unresolved issue is the distinction between genuine mechanical impairments and measurement-related artifacts. Abnormal thoracic geometry may both restrict regional myocardial motion and adversely affect acoustic windows and speckle tracking. Comparison with less acoustic-window-dependent techniques, particularly CMR feature tracking, may help distinguish between these mechanisms, although the lower temporal resolution of CMR may represent an important limitation for the assessment of myocardial deformation.

Longitudinal and interventional studies are particularly important for establishing causality. The reversibility of strain abnormalities after delivery in healthy pregnancy and the improvement in cardiac functional indices following surgical correction of PE [135,136,137] are consistent with a potentially reversible mechanical component. Future studies should determine whether changes in thoracic dimensions or relief of cardiac constraint are quantitatively paralleled by normalization of global and regional strain and whether these relationships persist after accounting for simultaneous changes in loading conditions, cardiac position, and image acquisition.

The regional distribution of these abnormalities also warrants investigation. Preferential basal and mid-ventricular impairment with relative apical preservation may represent a reproducible phenotype of thoracic constraint and potentially mimic patterns associated with intrinsic myocardial disease. Standardized segmental strain analysis combined with thoracic morphometry could determine whether such regional signatures improve differential diagnosis.

Intrinsic and extrinsic mechanisms may also coexist, as exemplified by MVP, where valvular–ventricular mechanical forces and restrictive thoracic geometry may jointly contribute to basal inferolateral strain impairment. Similarly, in pregnancy and obesity, major hemodynamic, metabolic, inflammatory, and body-composition determinants may coexist with changes in thoracic geometry. Future studies should therefore quantify the relative contributions of myocardial, hemodynamic, metabolic, body-composition-related, and extracardiac mechanical factors rather than attributing abnormal strain to a single mechanism.

Finally, methodological standardization and external validation are essential. Standardized A–P diameter and MHI acquisition, reproducibility testing, comparison with computed tomography (CT)- or CMR-derived thoracic dimensions, and validation across ultrasound platforms should precede routine implementation. Most importantly, prospective studies should determine whether incorporating thoracic morphometry into strain interpretation reduces false-positive classification of subclinical myocardial dysfunction and improves selection of patients requiring further diagnostic evaluation.

Overall, independent multicenter and multi-vendor validation, demonstration of causality, and definition of reproducible regional deformation patterns will be necessary before thoracic morphometry can be incorporated into routine strain interpretation and used to distinguish abnormal myocardial deformation from abnormal myocardium itself. At present, the proposed morphometric framework should therefore be considered hypothesis-generating rather than clinically validated. In parallel, greater transparency regarding myocardial strain algorithm behavior and its potential sensitivity to dynamic changes in ultrasound beam angulation throughout the cardiac cycle is warranted. These technical dependencies should be more extensively investigated and reported across manufacturers and vendors, ideally through standardized experimental settings using dedicated cardiac phantoms.

## 14. Conclusions

Myocardial strain should not be interpreted as an isolated surrogate of intrinsic myocardial contractility. Emerging evidence indicates that the mechanical environment surrounding the heart, particularly thoracic geometry and reduced A–P thoracic dimensions, may contribute to variations in global and regional myocardial deformation even in the absence of overt structural heart disease. A recurring pattern of preferential basal and mid-ventricular impairment with relative preservation of apical mechanics further suggests that thoracic constraint may contribute to a recognizable extrinsically mediated deformation phenotype, potentially mimicking relative apical sparing. However, the available evidence remains predominantly observational, derives in part from selected single-center cohorts, and does not establish a causal relationship between thoracic geometry and myocardial strain abnormalities.

These observations have potential implications for clinical strain interpretation. An abnormal strain value establishes the presence of abnormal deformation, but not its biological origin. When reduced LV-GLS or disproportionate basal impairment occurs despite otherwise preserved cardiac structure and function, thoracic geometry may therefore represent an additional contextual factor to be considered alongside technical, hemodynamic, and intrinsic myocardial determinants. Simple measurement of the A–P thoracic diameter, complemented when appropriate by the MHI, may provide a readily accessible mechanical context marker, but should not be regarded as a diagnostic endpoint or used to exclude myocardial disease. At present, these morphometric parameters should be considered exploratory rather than validated diagnostic markers.

The central implication is a shift from a numerical to a mechanistic interpretation of myocardial strain: the clinically relevant question is not only whether strain is abnormal, but why and where deformation is reduced. Integrating regional strain patterns with conventional cardiac findings, clinical probability, and thoracic morphology may ultimately improve diagnostic specificity and help distinguish abnormal myocardial deformation from abnormal myocardium itself. Independent multicenter and multi-vendor validation is required before this hypothesis-generating framework can be incorporated into routine clinical decision-making. Equally important, greater transparency regarding strain algorithm behavior and its sensitivity to dynamic changes in ultrasound beam angulation throughout the cardiac cycle is needed to better understand the technical contribution to measured deformation abnormalities.

## Figures and Tables

**Figure 1:**
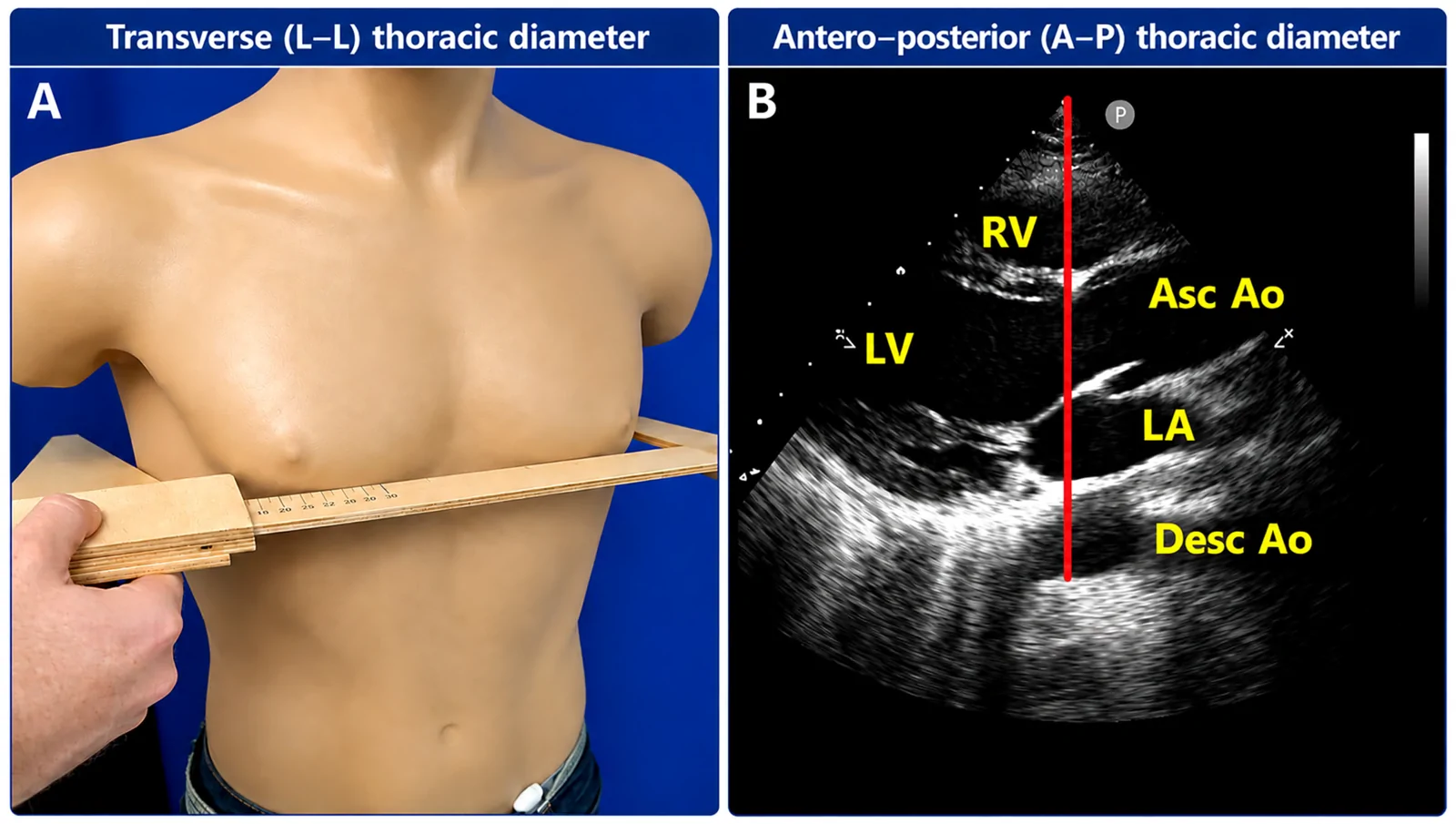
Anthropometric and echocardiographic assessment of thoracic geometry for calculation of the modified Haller index. (**A**) Measurement of the transverse (L–L) thoracic diameter using a dedicated centimeter-calibrated anthropometric device positioned across the anterior chest wall. (**B**) Measurement of the antero-posterior (A–P) thoracic diameter from the conventional parasternal long-axis echocardiographic view, as the distance between the ultrasound transducer entry point at the anterior chest wall and the anterior surface of the vertebral body (red line). The anterior surface of the vertebral body is identified at the level of the posterior wall of the descending thoracic aorta, which is visualized immediately posterior to the left atrium. The modified Haller index (MHI) is calculated as the ratio between the L–L and A–P thoracic diameters (MHI = L–L/A–P), providing a simple, non-radiological quantitative assessment of thoracic conformation. A–P, antero-posterior; Asc Ao, ascending aorta; Desc Ao, descending aorta; LA, left atrium; L–L, latero-lateral; LV, left ventricle; MHI, modified Haller index; RV, right ventricle. AI disclosure: ChatGPT (OpenAI, San Francisco, CA, USA; GPT-5.6 Sol) was used exclusively for graphical refinement of this figure. The scientific content and interpretation were determined and verified by the authors.

**Figure 2:**
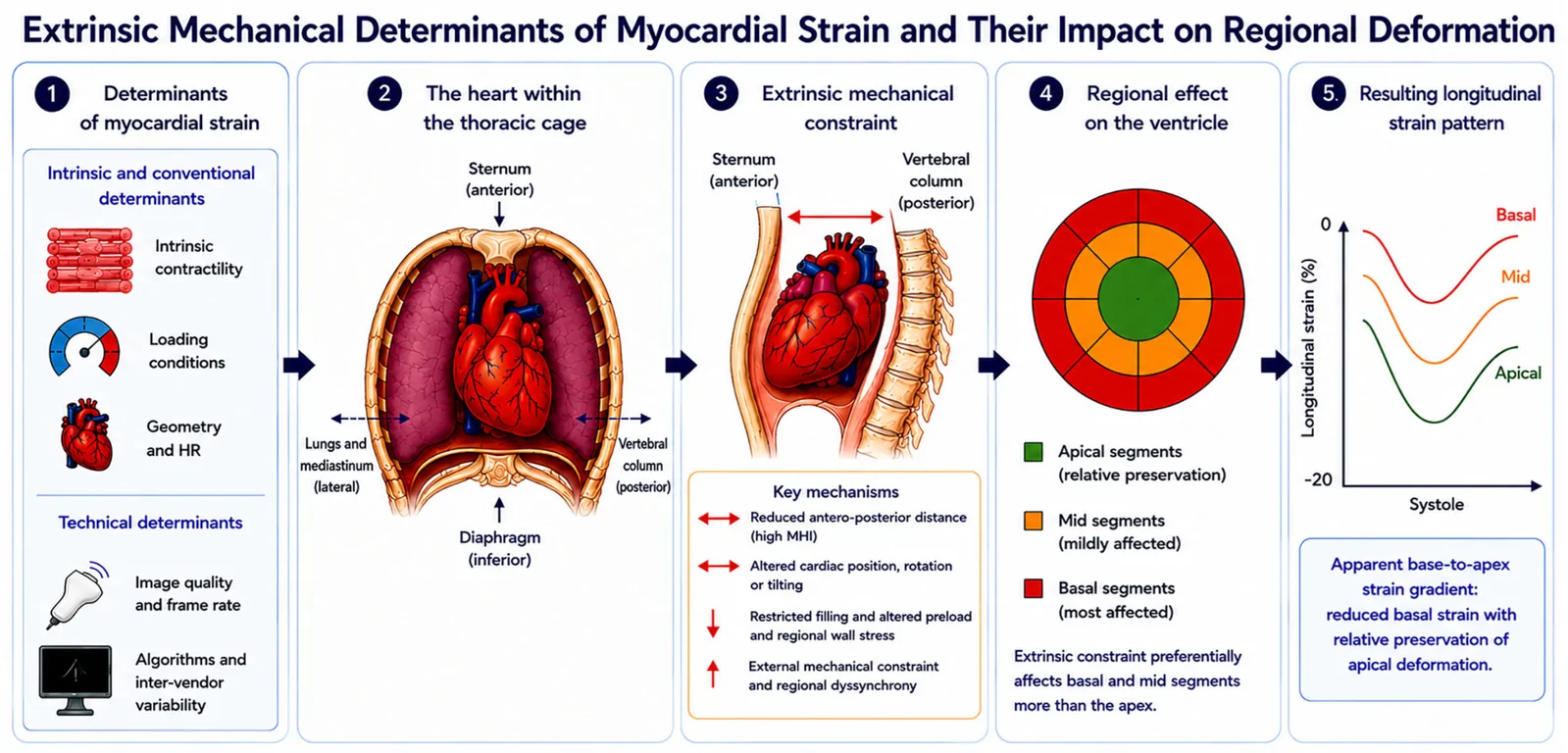
Extrinsic mechanical determinants of myocardial strain and their potential impact on regional deformation. Myocardial strain is influenced not only by intrinsic contractility and conventional physiological, hemodynamic, and technical determinants, but also by the mechanical environment surrounding the heart. A reduced antero-posterior (A–P) thoracic diameter decreases the sterno-vertebral space available for cardiac motion and may increase external mechanical constraint, preferentially affecting the basal and mid-ventricular segments while relatively preserving apical deformation. This regional distribution may generate an apparent base-to-apex strain gradient, characterized by reduced basal strain with relative apical preservation. Such a pattern may reflect a combination of true mechanically constrained myocardial deformation and measurement-related effects rather than primary myocardial disease. A–P, antero-posterior; HR, heart rate; MHI, modified Haller index. AI disclosure: ChatGPT (OpenAI, San Francisco, CA, USA; GPT-5.6 Sol) was used exclusively for graphical refinement of this figure. The scientific content and interpretation were determined and verified by the authors.

**Figure 3:**
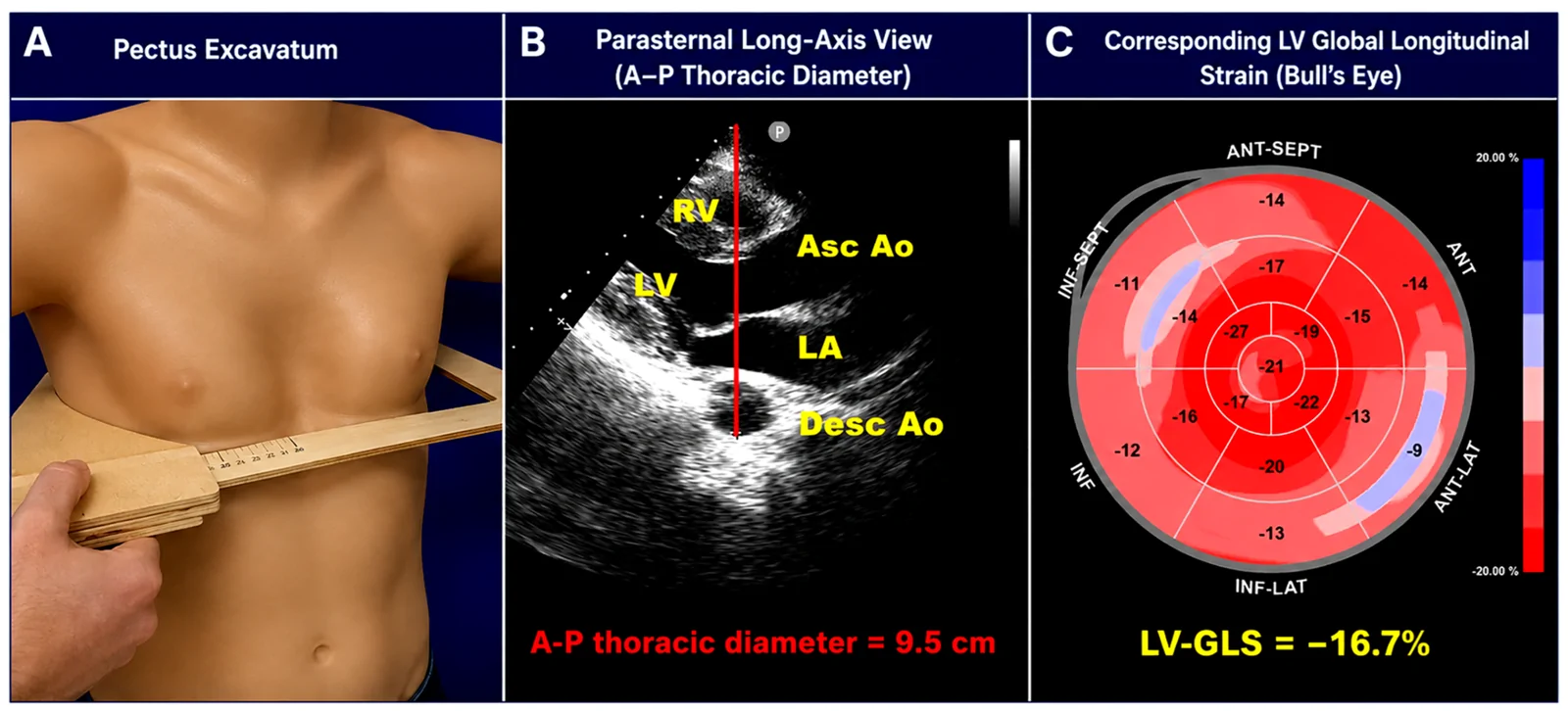
Integrated assessment of thoracic morphology and myocardial deformation in pectus excavatum. (**A**) Representative individual with PE undergoing measurement of the transverse (L–L) thoracic diameter using a centimeter-calibrated anthropometric device. (**B**) Measurement of the antero-posterior (A–P) thoracic diameter (9.5 cm) from the conventional parasternal long-axis echocardiographic view, used together with the L–L diameter for calculation of the MHI. (**C**) Corresponding LV longitudinal strain bull’s-eye obtained by STE, showing mildly reduced global longitudinal strain (LV-GLS, −16.7%) predominantly driven by impaired basal deformation, with relative preservation of apical strain. This example illustrates the integration of thoracic morphometry and myocardial deformation imaging for the mechanistic interpretation of reduced strain in PE. A–P, antero-posterior; Asc Ao, ascending aorta; Desc Ao, descending aorta; LA, left atrium; L–L, latero-lateral; LV, left ventricle; LV-GLS, left ventricular global longitudinal strain; MHI, modified Haller index; PE, pectus excavatum; RV, right ventricle; STE, speckle-tracking echocardiography. AI disclosure: ChatGPT (OpenAI, San Francisco, CA, USA; GPT-5.6 Sol) was used exclusively for graphical refinement of this figure. The scientific content and interpretation were determined and verified by the authors.

**Figure 4:**
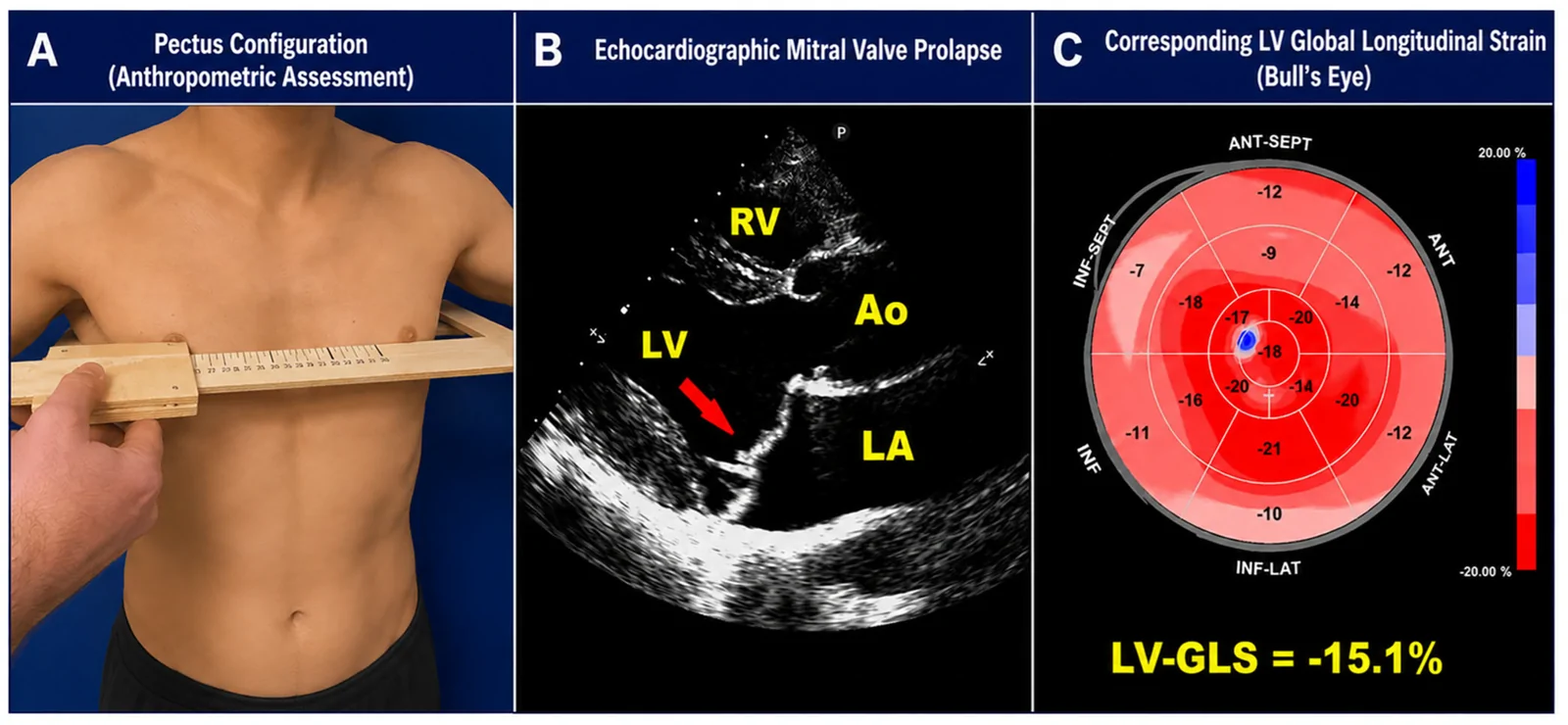
Thoracic morphology, mitral valve prolapse, and associated regional left ventricular longitudinal strain pattern. (**A**) Anthropometric assessment of thoracic geometry using a centimeter-calibrated device for measurement of the transverse (L–L) thoracic diameter. (**B**) Parasternal long-axis echocardiographic view demonstrating mitral valve prolapse, with systolic displacement of the mitral leaflet into the left atrium (red arrow). (**C**) Corresponding LV longitudinal strain bull’s-eye showing reduced LV-GLS (−15.1%), predominantly driven by attenuated basal segment deformation, with relative preservation of mid-ventricular and apical strain. The combined assessment illustrates how thoracic morphology may provide an additional mechanical context for interpreting regional myocardial deformation abnormalities in individuals with MVP. Ao, aorta; LA, left atrium; L–L, latero-lateral; LV, left ventricle; LV-GLS, left ventricular global longitudinal strain; MVP, mitral valve prolapse; RV, right ventricle. AI disclosure: ChatGPT (OpenAI, San Francisco, CA, USA; GPT-5.6 Sol) was used exclusively for graphical refinement of this figure. The scientific content and interpretation were determined and verified by the authors.

**Figure 5:**
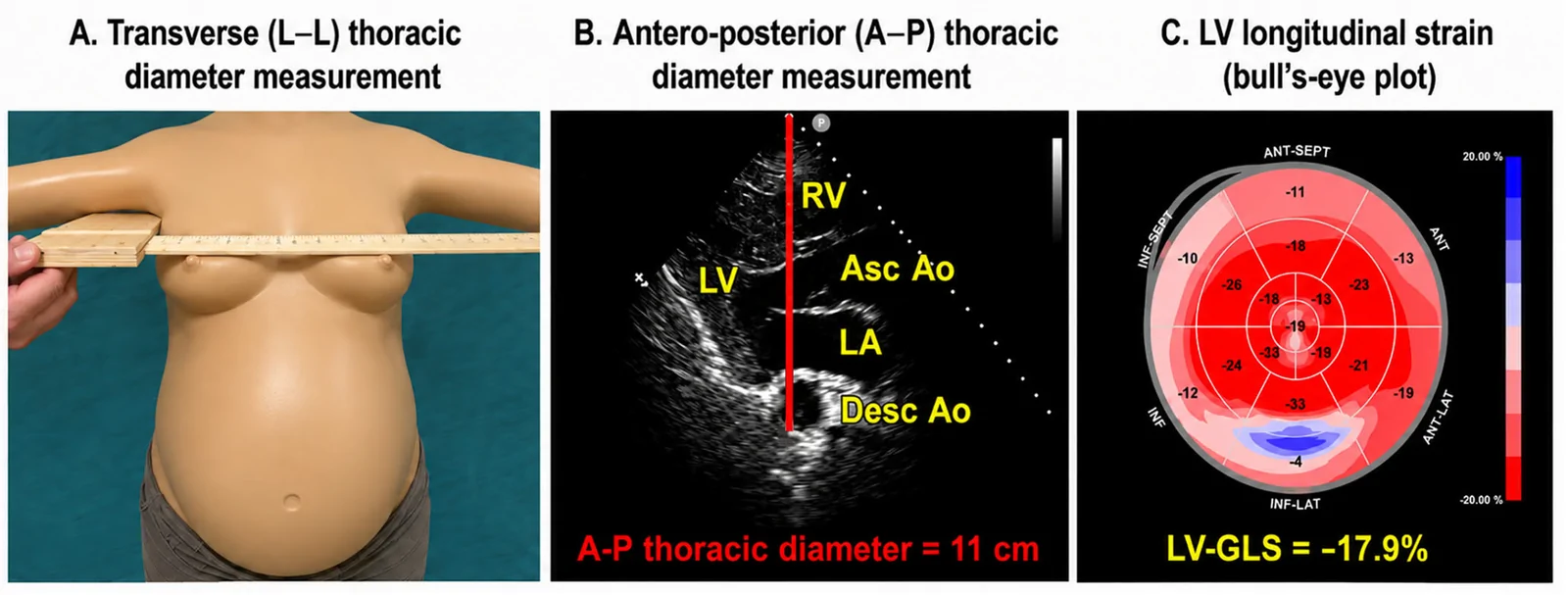
Integrated assessment of thoracic geometry and myocardial deformation in a healthy woman during the third trimester of pregnancy. (**A**) Measurement of the transverse (L–L) thoracic diameter using a centimeter-calibrated anthropometric device. (**B**) Measurement of the antero-posterior (A–P) thoracic diameter (11 cm) from the conventional parasternal long-axis echocardiographic view, used together with the L–L diameter for calculation of the modified Haller index (MHI). (**C**) Corresponding LV longitudinal strain bull’s-eye obtained by speckle-tracking echocardiography, showing mildly reduced global longitudinal strain (LV-GLS, −17.9%), predominantly driven by impaired deformation of the basal myocardial segments, with relative preservation of mid-ventricular and apical strain. This example illustrates the potential relationship between restrictive thoracic geometry and the characteristic base-to-apex deformation gradient observed during late pregnancy. A–P, antero-posterior; Asc Ao, ascending aorta; Desc Ao, descending aorta; LA, left atrium; L–L, latero-lateral; LV, left ventricle; LV-GLS, left ventricular global longitudinal strain; MHI, modified Haller index; RV, right ventricle. AI disclosure: ChatGPT (OpenAI, San Francisco, CA, USA; GPT-5.6 Sol) was used exclusively for graphical refinement of this figure. The scientific content and interpretation were determined and verified by the authors.

**Figure 6:**
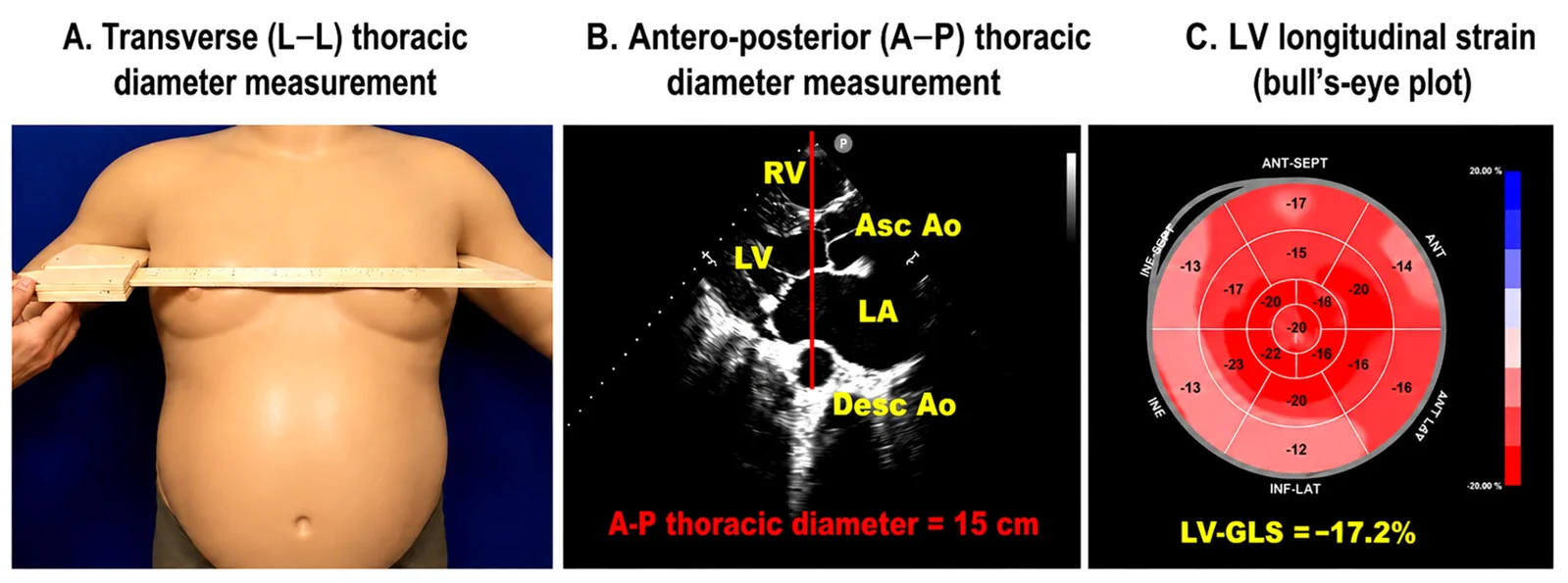
Integrated assessment of body geometry, thoracic morphology, and myocardial deformation in a healthy individual with obesity. (**A**) Healthy individual with obesity undergoing measurement of the transverse (L–L) thoracic diameter using a centimeter-calibrated anthropometric device. (**B**) Measurement of the antero-posterior (A–P) thoracic diameter (15 cm) from the conventional parasternal long-axis echocardiographic view, used together with the L–L diameter for calculation of the MHI. (**C**) Corresponding LV longitudinal strain bull’s-eye obtained by STE, showing mildly reduced global longitudinal strain (LV-GLS, −17.2%) predominantly driven by impaired basal deformation, with relative preservation of mid-ventricular and apical strain. This example illustrates how the integration of body and thoracic morphometry with myocardial deformation imaging may aid the mechanistic interpretation of reduced strain in an otherwise healthy individual with obesity. A–P, antero-posterior; Asc Ao, ascending aorta; Desc Ao, descending aorta; LA, left atrium; L–L, latero-lateral; LV, left ventricle; LV-GLS, left ventricular global longitudinal strain; MHI, modified Haller index; RV, right ventricle; STE, speckle-tracking echocardiography. AI disclosure: ChatGPT (OpenAI, San Francisco, CA, USA; GPT-5.6 Sol) was used exclusively for graphical refinement of this figure. The scientific content and interpretation were determined and verified by the authors.

**Figure 7:**
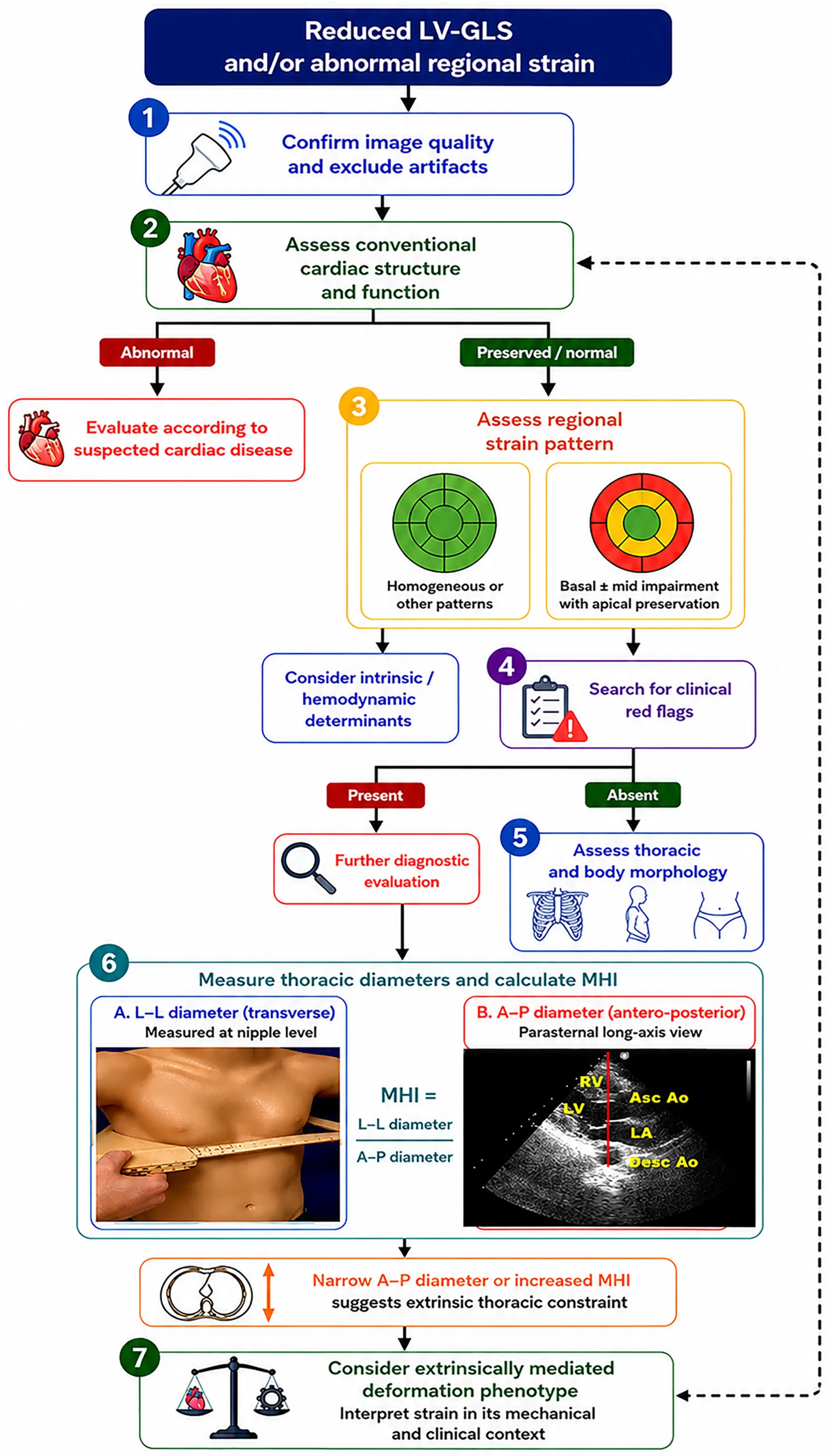
Proposed mechanistic algorithm for the interpretation of reduced myocardial strain in individuals without overt structural heart disease. Following confirmation of adequate image quality and exclusion of major technical determinants of strain variability, conventional cardiac structure and function and the regional distribution of myocardial deformation should be evaluated. Predominant basal and/or mid-ventricular strain impairment with relative apical preservation, particularly in the absence of clinical or imaging red flags for myocardial disease, should prompt consideration of extracardiac mechanical determinants. Assessment of thoracic morphology and measurement of the antero-posterior (A–P) thoracic diameter from the conventional parasternal long-axis view may identify a restrictive thoracic configuration. When appropriate, the modified Haller index (MHI) can provide additional quantitative characterization. Identification of a narrow A–P thoracic diameter or increased MHI should not exclude intrinsic myocardial disease but may support an extrinsic mechanical contribution to the observed strain abnormality. The dashed feedback arrow from Step 7 to Step 2 emphasizes that identification of a possible extrinsically mediated deformation phenotype should not be considered a definitive diagnostic endpoint; rather, strain findings should remain integrated with conventional cardiac structure and function and be reassessed if additional clinical or imaging abnormalities emerge. GLS, global longitudinal strain; L–L, latero-lateral; LV, left ventricular; MHI, modified Haller index. AI disclosure: ChatGPT (OpenAI, San Francisco, CA, USA; GPT-5.6 Sol) was used exclusively for graphical refinement of this figure. The scientific content and interpretation were determined and verified by the authors.

**Table 1:** Summary of the principal studies [31,32,61,90,107] evaluating thoracic morphology and myocardial deformation across the clinical models discussed in this review.

Clinical Model [Ref.]	Design/Population	Strain Assessment	Thoracic Assessment	Main Findings	Main Limitations
**PE, adults [31]**	Prospective case–control; 30 PE, 30 controls	2D-STE; Philips/QLAB	MHI; A–P, L–L diameters	Reduced LV and RV strain; strong relationship with MHI; predominant basal impairment	Single center; small selected cohort; no severe PE; single vendor
**PE, infants [32]**	Prospective case–control; 16 PE, 44 controls	2D-STE; Philips/QLAB	MHI; A–P, L–L diameters	Reduced biventricular strain; strong relationship with MHI maintained at short follow-up	Small PE cohort; short follow-up; no severe PE; single center/vendor
**MVP [61]**	Prospective case–control; 60 MVP, 60 controls	2D-STE; Philips/QLAB	MHI; A–P, L–L diameters	Reduced global/regional LV strain, particularly with restrictive thoracic conformation; basal predominance	Selected MVP cohort; no CMR reference; competing intrinsic MVP mechanisms; single center/vendor
**Pregnancy [90]**	Prospective longitudinal; 50 healthy women	2D-STE; Philips/QLAB	MHI; A–P, L–L diameters	Reversible strain reduction, mainly with restrictive thoracic conformation; relationship with MHI	Small cohort; gestational hemodynamic confounding; MHI not specifically validated in pregnancy; single center/vendor
**Obesity [107]**	Prospective case–control; 100 obesity, 60 controls	2D-STE; Philips/QLAB	HI/MHI; A–P, L–L diameters; waist circumference	Reduced LV-GLS; A–P diameter and waist circumference independently associated with impaired GLS	Selected cohort; CXR rather than CT reference; metabolic confounding; no external validation; single center/vendor

The available evidence is predominantly derived from observational, single-center studies, several conducted by the same research group; therefore, the reported morphometric thresholds (MHI > 2.5 for restrictive thoracic conformation [31,32,61,90] and nonradiological A–P thoracic diameter ≤ 17 cm in obesity [107]) should be considered exploratory rather than diagnostic. A–P, antero-posterior; CMR, cardiovascular magnetic resonance; CXR, chest X-ray; GLS, global longitudinal strain; HI, Haller index; L–L, latero-lateral; LV, left ventricular; MHI, modified Haller index; MVP, mitral valve prolapse; PE, pectus excavatum; RV, right ventricular; STE, speckle-tracking echocardiography.

## Data Availability

Data extracted from included studies will be publicly available on Zenodo (https://zenodo.org).
